# Pharmacists’ confidence when providing pharmaceutical care on anticoagulants, a multinational survey

**DOI:** 10.1007/s11096-017-0551-2

**Published:** 2017-11-14

**Authors:** John Papastergiou, Nadir Kheir, Katerina Ladova, Silas Rydant, Fabio De Rango, Sotiris Antoniou, Reka Viola, Maria Dolores Murillo, Stephane Steurbaut, Filipa Alves da Costa

**Affiliations:** 1 Leslie Dan Faculty of Pharmacy, University of Toronto, Toronto, ON M4J 1L2 Canada; 2 School of Pharmacy, University of Waterloo, 755 Danforth Avenue, Toronto, ON M4J 1L2 Canada; 3 College of Pharmacy, Qatar University, PO Box 2713, Doha, Qatar; 4 Department of Social and Clinical Pharmacy, Faculty of Pharmacy in Hradec Kralove, Charles University, Akademika Heyrovskeho 1203, 500 05 Hradec Kralove, Czech Republic; 5 Pharmaceutical Care Division (Meduca), Royal Pharmacists Association of Antwerp (KAVA), Lange Leemstraat 187, 2018 Antwerpen, Belgium; 6 Shoppers Drug Mart 1271, 2501 Third Line, Oakville, ON L6M 5A9 Canada; 7 Barts Health Centre; Barts Health NHS Trust, London, UK; 8 UCL Partners, London, UK; 9 Department of Clinical Pharmacy, Faculty of Pharmacy, University of Szeged, Szikrautca 8, Szeged, 6724 Hungary; 10 Farmacia Fernández Vega C.B., C/Par nº 26 Urbanización Club de Golf. Alcalá de Guadaira, Sevilla, 41500 Spain; 11 Research Group Clinical Pharmacology& Clinical Pharmacy (KFAR), Faculty of Medicine and Pharmacy, Vrije Universiteit Brussel, Laarbeeklaan 101, 1090 Brussels, Belgium; 12 Centro de Investigação Interdisciplinar Egas Moniz (CiiEM), Campus Universitário, Quinta da Granja, Monte da Caparica, 2829-551 Caparica, Portugal; 13 Portuguese Pharmaceutical Society (PPS), Rua da Sociedade Farmacêutica, 18, 1169-075 Lisboa, Portugal

**Keywords:** Anticoagulants, Education, pharmacy, iPACT, Knowledge, Needs assessment, Pharmacists

## Abstract

*Background* Guidelines on the management of orally anticoagulated patients are continuously evolving, leading to an increased need for pharmacists to be fully integrated in care provision. *Objective* To identify self-reported gaps in confidence among practicing pharmacists in the area of anticoagulation. *Setting* Pharmacists in different work settings in different countries. *Method* Cross-sectional international survey from October 2015 till November 2016 among pharmacists working in different settings to assess their level of confidence when delivering anticoagulants as well as to identify possible educational needs regarding this medication class. Validation of the survey was ensured. *Results* Responses from 4212 pharmacists originating from 18 countries were obtained. Pharmacists’ level of confidence was significantly higher (*p* < 0.001) when advising patients on vitamin K antagonists (VKAs) versus non-vitamin K antagonists (NOACs). In general, hospital pharmacists displayed higher confidence levels compared to community pharmacists when advising patients on anticoagulation (*p* < 0.001). Two distinct patterns of confidence levels emerged relating to basic and advanced pharmaceutical care. Confidence levels when providing advanced pharmaceutical care were significantly higher for Oceania and lower for South America (*p* < 0.005). *Conclusions* Pharmacists felt more confident in supporting patients receiving VKAs compared to the more recently introduced NOACs. With the increasing use of NOACs and the risks pertaining to anticoagulation therapy, it is essential to invest in education for pharmacists to address their knowledge gaps enabling them to confidently support patients receiving oral anticoagulants.

**Electronic supplementary material:**

The online version of this article (10.1007/s11096-017-0551-2) contains supplementary material, which is available to authorized users.

## Impact of findings on practice

- Education of pharmacists in anticoagulation is essential for improved pharmaceutical patient care
- Pharmacists perceive a greater need for education on more recently marketed anticoagulants
- Pharmacists identify e-learning as the preferred method of educational content delivery

## Introduction

Venous thromboembolism (VTE) and atrial fibrillation (AF) are two health conditions that require complex anticoagulation therapy for management and for prevention of life-threatening thromboembolic events. It is estimated that 10 million new cases of VTE are diagnosed annually resulting in over 544,000 deaths per year in Europe and 300,000 deaths per year in the US [1–3]. As 60% of VTE cases occur after hospitalization, it is a leading preventable cause of hospital-related death [1]. Globally, there are 33.5 million patients afflicted with AF [4]. It has been reported that as many as 10–27% of patients with AF remain undiagnosed [5]. AF can lead to ischemic stroke, and patients with AF are at a five-fold increased risk of stroke compared to the general population [6]. Depending on an individual’s CHADS_2_ score, the estimated risk of stroke ranges from 1.9 to 18.2% if a patient with AF does not receive anticoagulation [7]. In the US, 1 in 5 of the 700,000 strokes which occur annually are attributed to AF, and this proportion increases to 1 in 3 in those aged over 80 years [8]. Ultimately, patients with AF have a two-fold increased risk of mortality compared to the general population [6].

Oral anticoagulants are the standard modality for the treatment of these conditions. Compared to placebo, there is an 83% decrease in the recurrence of VTE with warfarin or non-vitamin K oral anticoagulant (NOAC) therapy [9]. Likewise, warfarin reduces the risk of ischemic stroke by 60% in patients with AF [10]. Comparatively, the NOACs have been shown to be either non-inferior or superior when compared to warfarin for stroke prophylaxis in AF [11–14].

Although warfarin was the most widely used OAC globally since its approval in 1954, the use of NOACs has steadily increased since the availability of dabigatran in 2008. In 2014, NOACs accounted for 15.5% of the global anticoagulant market whereas warfarin use had declined to 72% [15]. Furthermore, studies have estimated a three-fold increase in NOAC use between 2013 and 2014 in the United States (US) for AF [16]. This increase in use is likely attributed to the demonstrated safety, efficacy, and convenience of the newer agents, and the lack of international normalized ratio (INR) monitoring or individualized dose adjustments [11–14]. Nonetheless, patients are less likely to receive frequent follow-up as there is no standardized routine monitoring performed to assess the anticoagulation effects of the NOACs [17, 18]. While INR may serve as an indicator of patient adherence with warfarin, patient self-reported intake is the predominant adherence assessment used with NOACs [19]. A recent dabigatran analysis revealed that a 10% decrease in adherence resulted in a 13% increased stroke risk secondary to AF [20]. As such, patient monitoring, education, and medication adherence are of critical importance with these novel agents, given the difference in methods for assessing it as well as the consequences of non-adherence due to the NOACs’ shorter half-lives.

The scope of pharmacy practice continues to evolve globally, and pharmacists are uniquely positioned to play a broader role in anticoagulation medication management. Several studies have documented the positive impact from pharmacists in monitoring and managing warfarin in the community setting. These benefits include an increased percentage of INR measurements within therapeutic range, increased patient understanding of their medication, and reduced-rates of anticoagulation-related emergency room visits and hospitalizations [21–23]. Given that self-reported adherence rates with NOACs are as low as 57% [24], pharmacists may be able to have a similar impact on patients receiving NOAC therapy.

The International Pharmacists for Anticoagulation Care Taskforce (iPACT) is an expert group committed to further enhance the key role pharmacists play in anticoagulation management. Whilst pharmacists are well suited to monitor patients receiving oral anticoagulation, to our knowledge, an assessment of their knowledge in providing consultations has not been formally undertaken. Historically, needs assessment surveys have been successfully used to identify the self-perceived confidence of pharmacists in emerging therapeutic fields and to identify professional needs prior to developing continuing professional education and development programs [25, 26].

## Aim of the study

The main study purpose was to identify self-reported gaps in knowledge and confidence among pharmacists in the area of anticoagulation and to explore preferred educational methods to close these gaps. An additional objective was to identify any difference in confidence levels between countries.

## Ethics approval

This study was centrally approved by the Comissão de Ética Egas Moniz in Portugal (Process no. 489). In all countries, pharmacists were free to decline participation. The principles of ethical research practices were followed, such as confidentiality, freedom to participate or withdraw at any stage, and anonymity.

## Methods

### Design and procedure

This study was a cross-sectional international survey (available as electronic supplementary material) among pharmacists working in different settings to assess their level of confidence when delivering anticoagulants as well as to identify possible educational needs regarding this medication class. The questionnaire was originally developed and validated in English, following literature review, consultation with external experts and input from iPACT members experienced in survey development. The Decipher software (version M35; Quest Mindshare, Toronto, Canada) was used for developing the web-based survey; and data extraction was programmed on WinCross 14.0. The questionnaire consisted of three parts. The first part (including 6 items) collected participants’ demographic information such as years of work experience, level of education and area of practice. The second part (including 13 items) explored pharmacists’ confidence levels when advising patients on various aspects of anticoagulation medication, and was ranked on a 4 point Likert scale, ranging from “very confident” to “not confident at all”. The second part of the survey also asked about information sources sought to support patient counselling. The last part (including 6 items) gauged the perceived educational needs of the pharmacists and asked about the preferred format and tools to deliver this education. The survey was translated into 19 different languages and distributed in 22 countries after being piloted. Piloting was performed by administration to 10 pharmacists per country and to assess the burden, applicability, and acceptability among respondents. Subsequently, the first countries where data collection was undertaken (France and Canada), were used to validate the survey, which comprised testing psychometric properties in a sample of respondents. The process was then replicated in all participating countries. The survey was distributed electronically using each iPACT country coordinator’s network. The most common method involved using the national and/or regional professional pharmacy associations for dissemination. Since countries joined the project at different times, the survey was allowed to run from October 2015 until November 2016. In all countries, the national coordinator was free to decide on the length of data collection, which depended mainly on the partner associations involved in disseminating the survey and the time to reach the minimum number of 50 respondents per country. The minimum period to achieve this was 1 month and the maximum 6 months. In all countries, mail alerts were used as a reminder technique to increase the response rate.

### Statistical analysis

The survey’s construct validity was examined using factor analysis. Internal consistency and homogeneity were examined by calculating Cronbach’s alpha. Categorical data was summarized as frequencies and percentages. Comparison between groups was performed using the Chi square test while comparison of continuous variables was performed using independent sample *t* test or ANOVA. Depending on the number of groups under comparison, Bonferroni post hoc tests were utilized as required. Linear regression was performed to assess the effects of potential predictors on pharmacists’ confidence levels (dependent variable) when advising patients on anticoagulation medication. Predictors tested (independent variables) included age, years of experience, educational level, area of practice and country of origin. Statistical analyses were executed with a 0.05 significance level using IBM SPSS Statistics version 23 (IBM Corp., Armonk, NY, USA).

## Results

### Characterisation of respondents

**Fig. 1:**
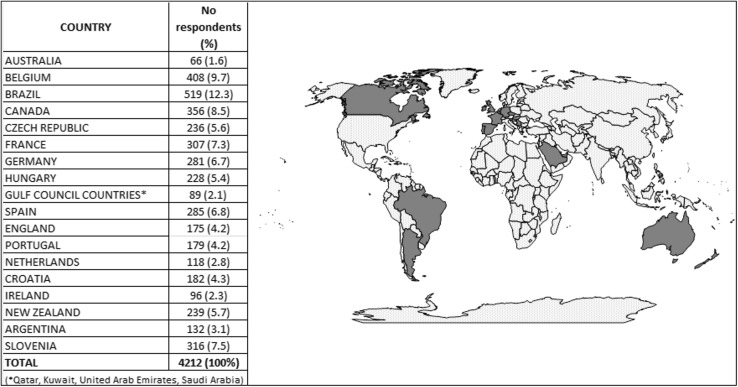
Distribution of respondents to the iPACT survey by country of origin

A total of 4255 responses from 21 countries were collected. Countries with no responses (Peru) or with a low response rate (USA, Slovenia and Chile) were subsequently excluded. As such, 18 countries with a total of 4212 responses were retained in the final analysis (Fig. 1). The final list of the countries included were representative of Europe (66.7%), North and South America (5.6 and 11.1%, respectively), Oceania (11.1%), and the Gulf Council Countries (5.6%).

The majority of respondents were female pharmacists (n = 3045; 72.3%), mostly middle aged between 30 and 50 years. Experience in pharmacy varied widely, unlike formal education where only a minority held a PhD. 67.7% (n = 2852) of respondents worked in a community pharmacy, while 27.5% (n = 1157) worked in a hospital. Other areas of employment, including academia, the pharmaceutical industry, and independent consultancy firms, represented a much lower proportion of the sample population.

### Confidence levels of pharmacists

**Fig. 2:**
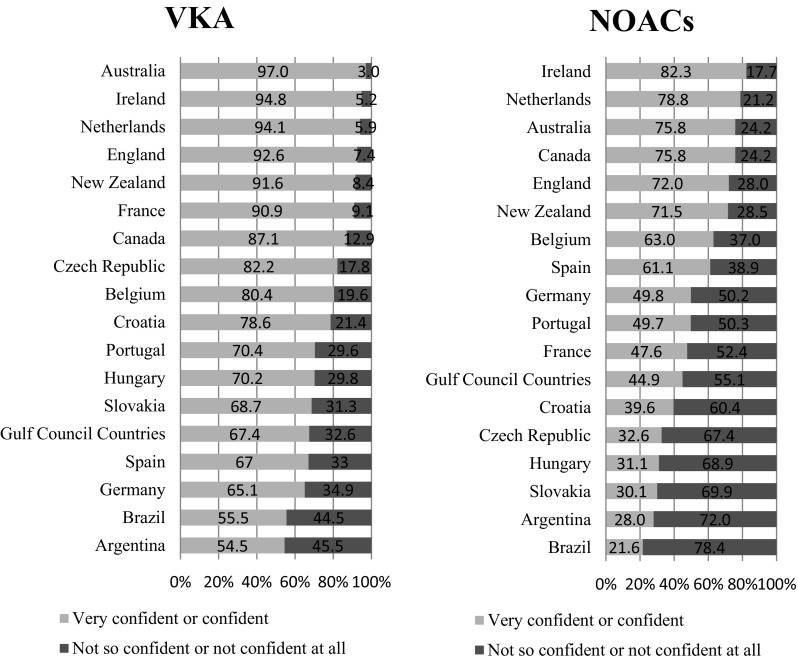
Distribution of pharmacists’ confidence by pharmacotherapeutic group

Pharmacists’ level of confidence was significantly higher when advising patients on vitamin K antagonists (VKAs) versus NOACs (*p* < 0.001) (Fig. 2).

**Fig. 3:**
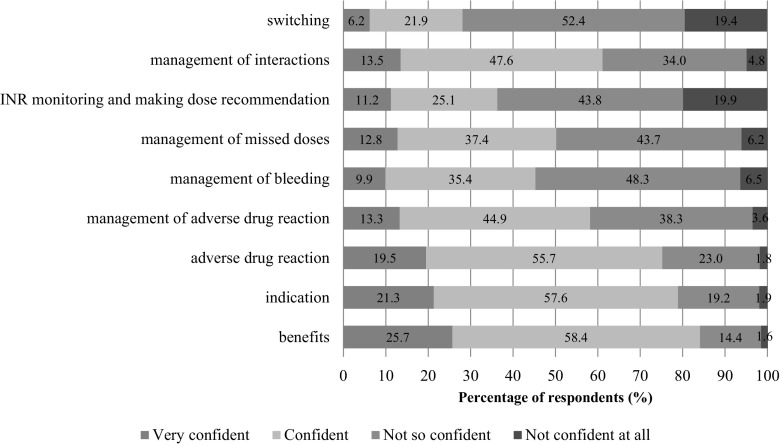
Distribution of pharmacists’ confidence by counselling item

Distinct patterns in confidence level were observed depending on the surveyed items (Fig. 3). There were no differences in the confidence levels that could be explained by the age of pharmacists, their experience of pharmacy practice or additional formal education (*p* > 0.05) (results not shown). In general, hospital pharmacists displayed higher confidence levels compared to community pharmacists when advising patients on anticoagulation (*p* < 0.001). However, country specific differences in confidence levels associated with area of practice were observed; for instance in Belgium, community pharmacists performed similarly to hospital pharmacists.

**Fig. 4:**
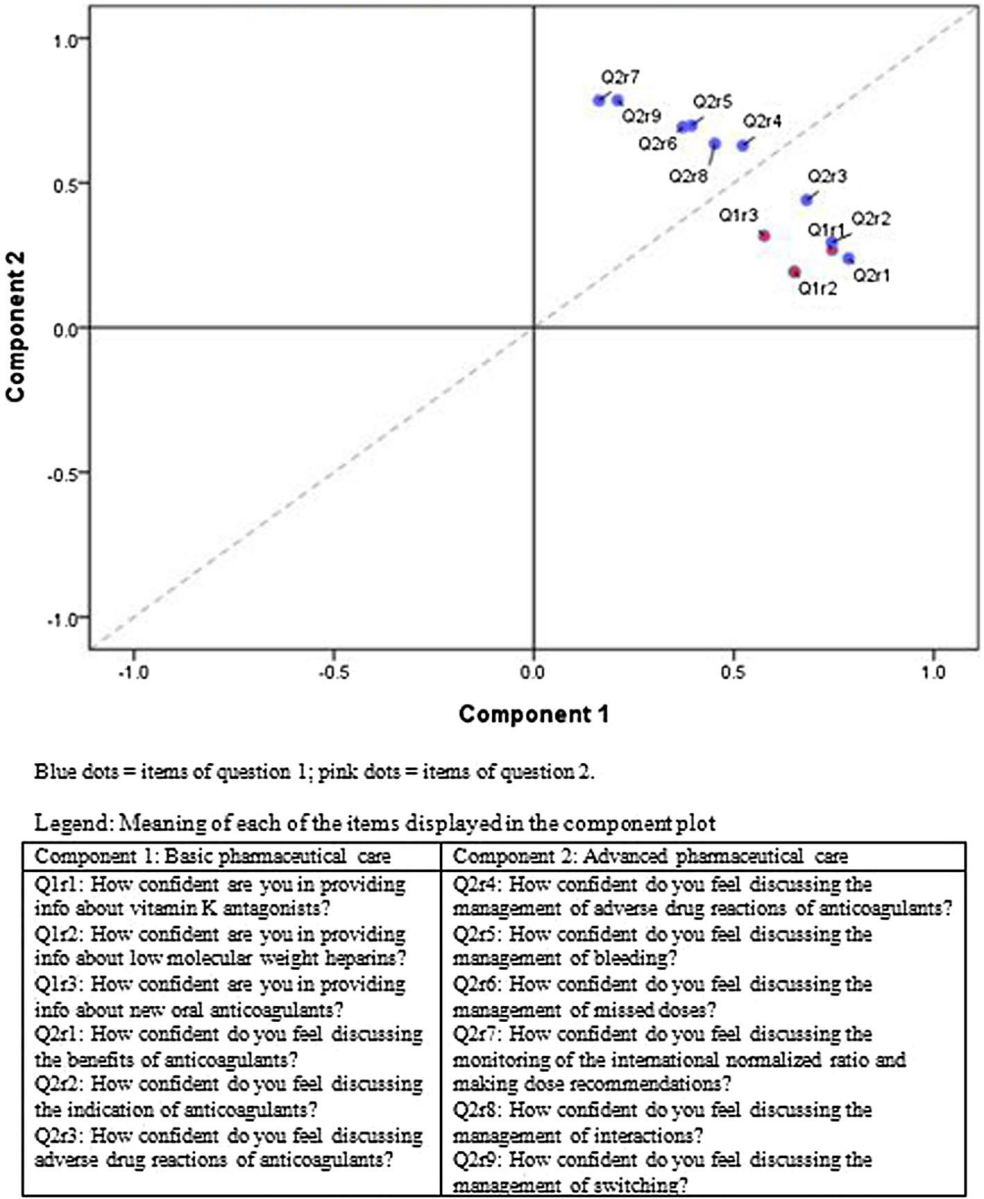
Component plot in rotated space

The underlying themes included in the questionnaire could be clustered into two main categories. The first group focused on delivering drug and disease-related information such as drug indications or expected benefits of treatment. In some countries, this practice is a compulsory part of basic pharmaceutical care. The second category included delivering more advanced pharmaceutical care and requiring the pharmacist to manage more complex issues such as the identification and treatment of bleeds. This categorization was confirmed by the rotated factor plotting, clearly visualizing that the individual items load into the respective components described (Fig. 4).

The two components shown in Fig. 4, basic pharmaceutical care and advanced pharmaceutical care, displayed high internal consistency in the overall sample (α = 0.851 and α = 0.877, respectively), with insignificant variations at the country level (ranging from 0.757 to 0.878 for component 1 and from 0.772 to 0.924 for component 2).

**Fig. 5:**
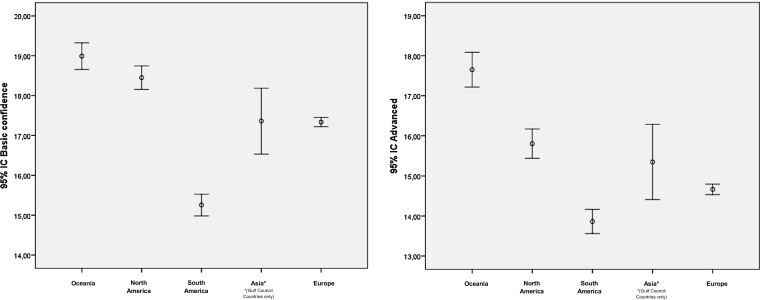
Comparison of confidence levels by continent of practice of pharmacists

Analysis of confidence levels by continent of origin identified differences between the groups for the two components (*p* < 0.001). Post-hoc analysis highlighted that with regard to confidence levels when providing patients with basic pharmaceutical care (component 1), only South American countries performed significantly lower than all others (*p* < 0.001). On the other hand, two extremes in confidence levels were detected when providing patients with advanced pharmaceutical care (component 2). South American countries performed significantly lower and Oceania performed significantly better (*p* < 0.005) in this regard compared to all others (Fig. 5).

Linear regression was used to analyse predictors of pharmacists’ confidence levels. Two independent models were considered, where component 1 and component 2 were the dependent variables. Whilst both models were found to predict very little of the variability (R^2^ = 0.196 and R^2^ = 0.233, respectively), the same variables were found to influence confidence levels, suggesting that gender, length of experience, post-graduate education, and area of practice may indeed partly explain self-confidence. On the other hand, continent of origin was identified as an additional predictor but only for component 2 (Table 1).

**Table 1:** Results from linear regression using components 1 and 2 on “confidence when advising patients on anticoagulation care” as the dependent variable (n = 4212)

	Adjusted β	95% CI	*p*-value
*Factor 1: Basic pharmaceutical care*			
Constant		18.318;19.991	< 0.001
Gender	− 0.159	− 1.399; − 0.953	< 0.001
Experience of pharmacy	0.096	0.138; 0.265	< 0.001
Additional education	0.041	0.023; 0.162	0.009
Area of practice	− 0.035	− 0.357; − 0.024	0.025
*Factor 2: Advanced pharmaceutical care*			
Constant		13.744; 15.618	< 0.001
Gender	− 0.157	− 1.560; − 1.060	< 0.001
Experience of pharmacy	0.084	0.128; 0.271	< 0.001
Additional education	0.083	0.271; 0.294	< 0.001
Area of practice	− 0.074	− 0.643; − 0.270	< 0.001
Continent	0.090	0.756; 1.511	< 0.001

### Preferred sources of information for pharmacists and educational needs

When dispensing anticoagulants, pharmacists chose specialized pharmacy software as their preferred source of information (n = 2350; 55.8%), followed by the internet (n = 2186; 51.9%). Less than half of the respondents used reference books (n = 1790; 42.5%) or published journal articles (n = 1255; 29.8%).

The majority of respondents (n = 3871; 91.9%) reported that they would like to receive additional education to improve their confidence level. The preferred areas for further education followed an inverse trend compared to the expressed confidence levels. The most desired item in this regard was interactions (n = 2956; 70.2%), followed by management of bleeding (n = 2871; 68.2%), and management of switching (n = 2780; 66.0%). Management of bleeding was among the top areas receiving educational requests, while learning about the coagulation pathway was the least requested topic (n = 1322; 31.4%).

The most popular formats of education were personalized e-learning (n = 2490; 59.1%), interactive websites (n = 1760; 41.8%) and symposia (n = 1757; 41.7%). The least preferred formats identified were education from peers (n = 493; 11.7%), webinars (n = 970; 23.0%) and workshops (n = 1473; 35.0%). While these results reflect the overall trend, there were country-specific behaviours, where for example education from peers was more popular among Dutch pharmacists and webinars amongst Croatians pharmacists.

In addition to education, pharmacists expressed the need to have a list of frequently asked questions to support them while counselling patients (n = 4043; 96.0%). A quick reference guide was proposed as a solution, either embedded in the pharmacy software (2754; 65.4%), as a link to a website (n = 2200; 52.2%) or as a specific app (n = 1797; 42.7%). Non-technological solutions were less favoured, e.g. the availability of a pocket guide.

## Discussion

To the best of our knowledge, this is the first global study that reports data from pharmacists on perceived gaps in confidence and training needs in the area of anticoagulation therapy. The diverse, transcontinental sample provides a valuable snapshot of the status of pharmacists with respect to their preparedness to provide counselling and education related to these potentially risky treatment modalities.

Non-vitamin K oral anticoagulants have been introduced into the market relatively recently as an alternative to warfarin, and our results showed a higher level of confidence among pharmacists advising patients on VKAs than on NOACs. This reflects the long experience with VKAs, which have been the mainstay of treatment globally for a much longer period. However, the complexity of therapies associated with VTE and AF requires an advanced level of practice, regardless of the anticoagulant used. Warfarin is consistently reported within the top 3 medications associated with an adverse reaction leading to a hospital admission [27]. Guidelines recommend that healthcare providers who are managing oral anticoagulation therapy should incorporate, in a coordinated manner, patient education, patient tracking and follow-up and good patient communication regarding the results and dosing decisions [28, 29]. For patients receiving VKA anticoagulation therapy, careful INR testing is key to ensuring treatment success, preventing thromboembolic events, and managing bleeding risks. Such an advanced level of practice is typically seen in countries known to have well-developed pharmacy practice standards and regulations that support those standards. The results with respect to pharmacist confidence levels with anticoagulation therapies mirrored this relationship. Respondents from Ireland, the UK, the Netherlands, Australia, Canada, and New Zealand expressed higher levels of confidence in dealing with VKAs and NOACs than respondents from the other countries. Respondents from South America were persistently less confident. Experience with NOACs and their use in routine practice differs between countries; and this may be influenced by health economics, pricing, and coverage by different health systems [28]. Higher gross domestic product (GDP) countries, for example, do tend to have advanced pharmacy practice in contrast with lower GDP nations [30]. This most likely explains the reason for the higher confidence illustrated in our results. Site of practice also has an association with the level of perceived confidence in dealing with anticoagulation management. In general, hospital pharmacists expressed higher confidence than pharmacists in other localities. This could be attributed to several factors. For example, the job demands in a hospital setting, the interprofessional environment in hospital practice, and the opportunity for professional development in many hospitals could explain this result. In contrast, community pharmacy in ‘developing’ countries is still dominated by the more traditional dispensing practice.

Over 90% of the participants expressed desire for some sort of continuing education on different aspects of anticoagulation management. Assessing training needs was an important objective in this study. While this was an opportunity for reflection from the perspective of the pharmacist, it helps in the development of relevant educational programs tailored to the perceived needs. The results of this study have also provided us with an insight on what information is desired, and in what format this information should be provided. Methods for learning spanned a wide range of options, with e-learning and interactive websites being the most popular. All this information should prove invaluable in developing continuous education materials to close the knowledge gaps identified by this study. Other studies using similar (web-based survey) or comparable techniques (interview, questionnaire) have also identified pharmacists’ knowledge gaps and education needs in domains such as transplantation, travel health, weight loss and oral health [31–34].

This study has some limitations, including the inability to include a larger sample in some relevant countries represented by iPACT members, like the USA or Chile, leading us to exclude them in the final analysis. Nonetheless, data collection is ongoing in these countries and may be used in the future to shape pharmacists’ education. The inability to represent African countries and include only Gulf Council countries (Kuwait, Qatar, Saudi Arabia and the United Arab Emirates) as representatives of the Asian continent limits comparisons. However, the similarities in pharmacy practice, demographics and education of Gulf Council countries allowed for a valid grouping of these individual countries [35]. Within participating countries, selection bias may have occurred by using an internet survey. However, in Portugal data was compared against national data of pharmacists’ demographics, and this showed only minor discrepancies [36]. The simplified translation process utilized may have led to some inadequate terminology use, but the high consistency evidenced led us to discard this possibility.

## Conclusion

Pharmacists reported higher confidence in supporting patients receiving VKAs compared to the more recently introduced NOACs. Consistently, pharmacists had greatest confidence in explaining the indication and the benefits of anticoagulation and lowest confidence in managing bleeding and switching amongst oral anticoagulants. With the increasing use of NOACs and the known risks pertaining to anticoagulation therapy, it is essential to invest in education for pharmacists to address their knowledge gaps enabling them to confidently support patients receiving oral anticoagulants.

## Electronic supplementary material

Below is the link to the electronic supplementary material.

Supplementary material 1 (DOC 64 kb)
