# Supplementary material for: Pharmacists’ confidence when providing pharmaceutical care on anticoagulants, a multinational survey
Source: Int J Clin Pharm. 2017 Nov 14;39(6):1282–90. doi: 10.1007/s11096-017-0551-2 (PMC5694509; doi:10.1007/s11096-017-0551-2)
Supplement: Supplementary file 1 — Supplementary material 1 (DOC 64 kb) [file 11096_2017_551_MOESM1_ESM.doc]

**TOOLS NEEDED FOR EFFECTIVE COUNSELLING AND DISPENSING OF ANTICOAGULANTS**

This survey has been developed by one of the iPACT Working groups to assess the pharmacists’ need for knowledge and skills (in several countries) when counselling patients who receive anticoagulant therapy. iPact (International Pharmacists for Anticoagulation Care Taskforce) is committed to improving pharmaceutical care for patients on oral anticoagulants. We really appreciate your help in answering the following questions. Completion of the survey should take no more than 5-10 minutes. All your answers shall remain strictly confidential and you are not required to reveal your identity. If you require any further information or assistance in completing the survey, you may contact Fabio De Rango at [fabderango@gmail.com](mailto:fabderango@gmail.com). By continuing the survey you are authorizing your anonymous data to be used for analysis and subsequent publication.

**A. What is your gender?**

- Male
- Female

**B. What is your age in years?**

- 18 – 29
- 30 – 39
- 40 – 50
- > 50

**C. How many years have you been practicing pharmacy? How many years have you been practicing pharmacy?**

o 5 or less

o 6 – 10

o 11 – 15

o 16 – 20

o >20

**D. What is your highest level of completed education?**

- Bachelor of Science
- Master
- Pharm D
- PhD
- Other: Please Specify:____________

**D. Where are you currently practicing pharmacy? Please select all that apply.**

- Hospital (in the outpatient pharmacy)
- Hospital (in wards)
- Specialized clinic (as independent advisor)
- Community (in the community pharmacy)
- GP-Practice (as independent advisor)
- Other: Please Specify: _______________

**F. In which country do you practice?**

- - Argentina
  - Australia
  - Austria
  - Belgium
  - Brazil
  - Canada
  - Chile
  - China
  - Croatia
  - Czech Republic
  - Dubai
  - England
  - France
  - Germany
  - Hungary
  - Italy
  - New Zealand
  - Oman
  - Qatar
  - Peru
  - Portugal
  - Slovakia
  - Slovenia
  - Spain
  - United Arab Emirates
  - United Kingdom
  - United States

**Please indicate the response that most accurately describes your confidence and needs in order to ensure that patients are treated appropriately with anticoagulation therapy:**

1. **How confident are you in providing the necessary information to patients receiving anticoagulation therapy?**

- **With vitamin k antagonists (VKA’s) such as warfarin and coumarins?**
  - Very confident
  - Confident
  - Not so confident
  - Not confident at all
- **With low molecular weight heparins (LMWH’s)?**
  - Very confident
  - Confident
  - Not so confident
  - Not confident at all
- **With direct acting oral anticoagulants (NOAC’s, DOAC’s) such as apixaban, dabigatran, edoxaban and rivaroxaban?**
  - Very confident
  - Confident
  - Not so confident
  - Not confident at all

1. **How confident do you feel in discussing the following with patients?**

- **Benefits of anticoagulation therapy?**
  - Very confident
  - Confident
  - Not so confident
  - Not confident at al**l**
- **The indications of anticoagulant therapy?**
  - Very confident
  - Confident
  - Not so confident
  - Not confident at all
- **The adverse effects of anticoagulant therapy?**
  - Very confident
  - Confident
  - Not so confident
  - Not confident at all
- **Management of adverse effects when it comes to anticoagulation therapy (including bleeding risk)?**
  - Very confident
  - Confident
  - Not so confident
  - Not confident at all
- **The management of bleeding while on anticoagulation therapy?**
  - Very confident
  - Confident
  - Not so confident
  - Not confident at all
- **How to manage missed doses?**
  - Very confident
  - Confident
  - Not so confident
  - Not confident at all
- **Monitoring INR and making dosing recommendations based on those values?**
  - - Very confident
    - Confident
    - Not so confident
    - Not confident at all
- **Management of interactions with anticoagulation therapy?**
  - Very confident
  - Confident
  - Not so confident
  - Not confident at all
- **Management of bridging/switching from one anticoagulant to another)?**
  - Very confident
  - Confident
  - Not so confident
  - Not confident at all

1. **What references do you use today in practice when dispensing anticoagulants?**
   - Company/industry literature
   - Internet
   - Reference books
   - Published journals
   - On line forums
   - Quick reference guides
   - Pharmacy software
   - Other reference: Please Specify: __________________
   - I do not use any reference
2. **Would you like to receive additional education in the area of anticoagulation?**
   - Yes
   - No
3. **If you replied yes to the previous question, what areas would interest you in receiving additional education in the area of anticoagulation (please select all that apply)?**

- Coagulation Pathway
- Medical conditions requiring anticoagulation therapy
- Mechanism of action of anticoagulants
  - Interactions (e.g. drug-drug; drug-food) with anticoagulants & their management
  - Possible side effects of anticoagulants & their management
  - How to assess risk of bleeding
  - How to manage bleeding while the patient is on anticoagulants
  - Bridging/switching
  - Other: Please Specify: ______________

1. **What form of education would you prefer (please select all that apply)?**
   - Personalized e-learning
   - Webinar
   - Interactive websites
   - Seminar/symposium
   - Workshop
   - From colleagues
   - Other: Please Specify**:** _______________
2. **Would a list of frequently asked questions be useful to include in material that would support you professionally?**

- Yes
- No

1. **What tools would you find useful as a quick reference when it comes to anticoagulation therapy (please select all that apply)?**
   - An application
   - A pocket guide
   - A desk manual for the pharmacy counter
   - A booklet
   - A (link to a) website
   - Information accessible from within pharmacy software
   - Other: Please Specify:_________________________________________________
2. **What would you consider important to be included on a quick reference (please select all that apply)?**
   - Indications
   - Dosing
   - Interactions
   - Side effects
   - Treatment of bleeding
   - Bridging/switching between different anticoagulants
   - Other: Please Specify:___________________________________________________
